# The effect of educational program based on theory of planned behavior on promoting retinopathy preventive behaviors in patients with type 2 diabetes: RCT

**DOI:** 10.1186/s12902-021-00680-2

**Published:** 2021-01-14

**Authors:** Seyed Shahriar Hosseini, Mohsen Shamsi, Mahboobeh Khorsandi, Rahmatollah Moradzadeh

**Affiliations:** 1 Department of Health Education and Promotion, Faculty of Health, Arak University of Medical Sciences, Arak, Iran; 2 Department of Epidemiology, Faculty of Health, Arak University of Medical Sciences, Arak, Iran

**Keywords:** Diabetes care, Health education, Investigation, Retinopathy, Theory of planned behavior

## Abstract

**Background:**

Diabetic retinopathy is the most common microvascular complication of diabetes and it is a leading cause of visual impairment and blindness among patients with diabetes. This study aimed to investigate the effect of educational program based on Theory of Planned Behavior (TPB) on promoting retinopathy preventive behaviors in patients with type 2 diabetes.

**Methods:**

The present study is an educational randomized controlled trial research that was conducted on 94 patients with type 2 diabetes who had gone to diabetes clinic. The samples were randomly assigned to the intervention (*N* = 47) and control groups (*N* = 47). Data collection instrument was a researcher-made questionnaire based on TPB and FBS and HbA1C tests. Then, educational program was performed for the intervention group through four educational sessions. After 3 months, data collection was repeated for the two groups and FBS, HbA1C testes were done again and data were analyzed.

**Results:**

The performance of the intervention group on preventive behaviors of retinopathy increased from 2.48 ± 1.42 to 4.48 45 1.45 after the education (*p* < 0.001). The mean of FBS and HbA1c in the intervention group also decreased after the intervention (*P* < 0.05).

**Conclusion:**

Applying the TPB model proved is very effective in developing an educational program for patients with diabetes, to control their blood sugar and enhance preventive behaviors of retinopathy. Besides such programs, follow-up education for controlling and monitoring are highly recommended. This theory serves as a helpful theoretical framework for health-related behaviors and can be an appropriate pattern to plan for educational interventions.

**Trial registration:**

This trial has been registered at Iranian Registry of Clinical Trials, IRCT20180819040834N1. Prospectively registered 8 Apr 2019, https://en.irct.ir/trial/38401

## Background

Diabetes, a major public health problem affecting more than four hundred million people worldwide [1]. According to a study conducted in 116 countries from 2010 to 2019, the prevalence of diabetes in adults aged 20 to 79 will increase from 6.9% in 2010 to 7.7% in 2030 [2].

Diabetes has been associated with the development of various complications including retinopathy [1, 3]. Studies have shown that people with diabetes are 25 times more likely to be blind than others [3, 4]. Optimal management of diabetic retinopathy should include annual screening, adequate control of associated risk factors and timely treatment [1, 5].

Currently, with the rising prevalence of diabetes in the world, WHO has declared it as a latent epidemic and believes that increasing patients’ awareness about complications disease [6]. A significant element towards an optimal management, which is often undervalued, is the improvement of knowledge and education among patients with diabetes [1]. Therefore, it is essential to have information about the beliefs and awareness of those at risk in order to develop preventive strategies [7].

Previous studies assessing knowledge, attitude and practices regarding eye diseases in patients with diabetes for example a study in Turkey showed that 31% of patients with diabetes had not received eye care training and did not know that the disease affects their eyesight [8]. Also in Nepal, only 12% of patients with diabetes were aware of the ocular complication of diabetes [9]. Other studies have emphasized the need to educate patients with diabetes to increase their awareness and performance in the prevention of retinopathy [5, 7, 10]. On the other hand, due to the important role of patients with diabetes in adopting health behaviors to prevent the complication of retinopathy, the importance of performing educational interventions based on appropriate behavioral theories for these patients is even greater. Therefore, in the present study, Theory of Planned Behavior (TPB) has been used.

**Fig. 1:**
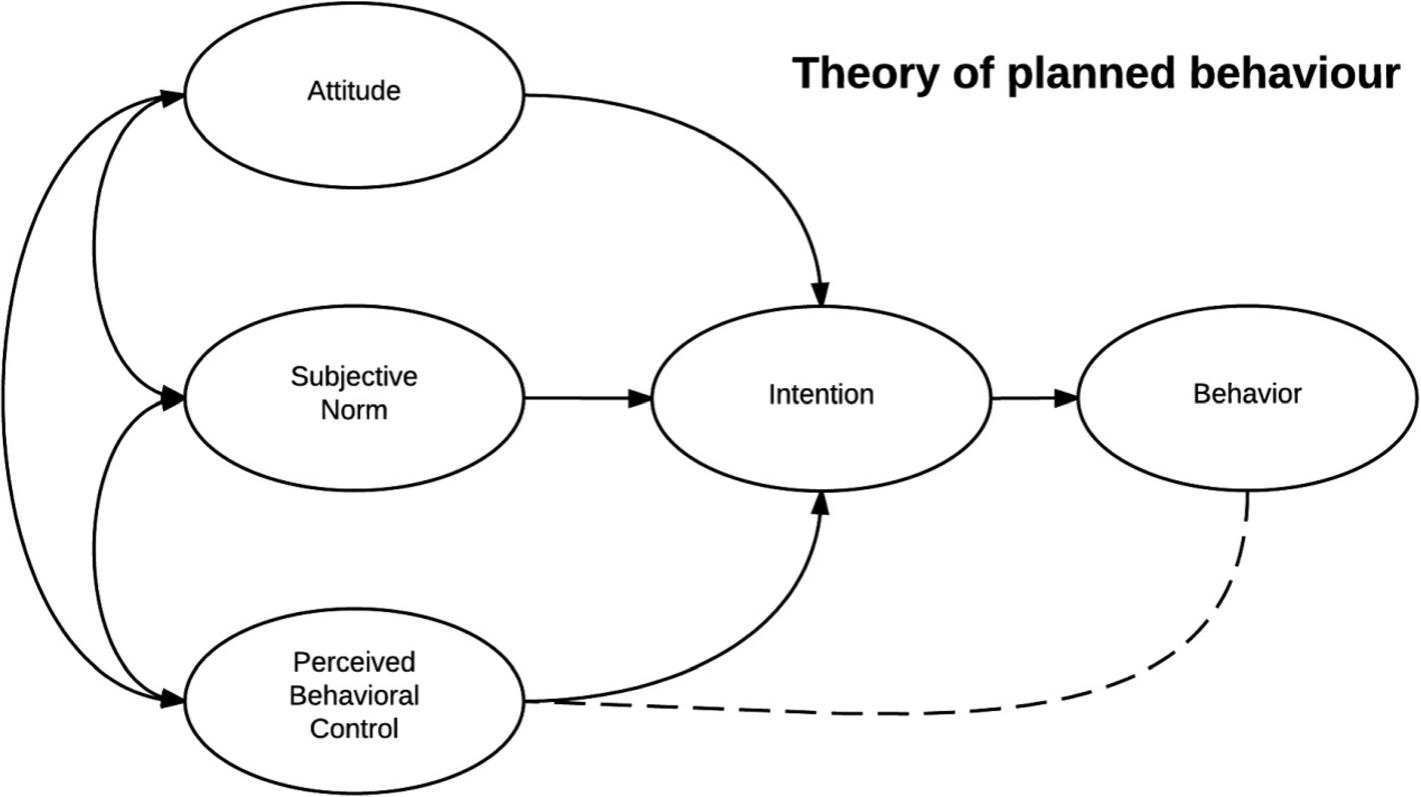
Theory of Planned Behavior (TPB). In this theory, by creating a positive attitude and promoting patients’ subjective norms towards the symptoms of retinopathy and eye care, along with perceived behavior control training and increasing their self-efficacy, eye care performance will improve in the patients

According to this theory, a patient’s attitude is his or her favorable or unfavorable evaluation to perform a particular behavior that has been formed through his or her mental perceptions or past experiences. Behavioral intention is the decision of an individual to adopt a behavior, and subjective norms are the effects of different people on the behavior of an individual. Perceived behavioral control refers to patients perception of his or her competence to successfully perform hygiene-related behaviors [11, 12]. Prevention care includes blood sugar control behaviors, regular visits to an ophthalmologist and timely eye examinations, adherence to a medication regimen, and adherence to a proper diet. To measure patients’ behavior more accurately Fasting Blood Sugar (FBS) and HbA1C quarterly blood sugar were used. Figure 1 shows Theory of Planned Behavior.

According to the studies, no intervention was found based on TPB on the promotion of eye care behaviors in patients with diabetes. Therefore, in the present study, it has been tried to teach eye care behaviors in patients with diabetes based on TPB constructs and to measure the effect of this training was assessed by measuring the behavior and blood sugar control indicators of FBS and HbA1C in the patients.

## Methods

This study is an educational randomized controlled trial (single blind) that was carried out on 94 patients with diabetes referred to Diabetes Clinic in Arak. Prospectively registered 5 Apr 2019, https://fa.irct.ir/trial/38401. This study adheres to CONSORT guidelines.

To determine the sample size based on similar study [13] a total of 42 individuals were calculated for each group, with 10% added to the sample size in each group, taking into account the rate of non-response to the questionnaires and the loss of samples during follow-up. Finally 47 individuals in each group were calculated in each group.

For sampling, a list of all patients was obtained from Diabetes Clinic of Arak. Then, 94 samples were selected by simple random sampling from patients with the criteria for entering the study and were randomly divided into two groups of control and intervention.

The conceptual framework of this study was that according to the similar study Malekmahmoodi et al. [14] about primary and secondary out-come and structure of intervention program based on TPB.

Inclusion criteria were patients with at least 1 year of diabetes history, no ocular complications, volunteering to participate in the study, being between 30and 70 years old, and literate at least until the fifth grade. Exclusion criteria included patients who developed ocular complications during the study at the discretion of the ophthalmologist and needed special treatment and educations, lack of patients willingness and refusing to participate in the study.

### Data collection tool

The data collection instrument was a valid and reliable questionnaire that was previously used in a studies [13, 14] consisted of the following sections:

1. Patient Demographic Information Questionnaire including age, occupation, education, duration of the disease, and type of treatment.
2. Patient Awareness Questionnaire for Diabetes and Diabetic Complications, which included 10 four-choice items.
3. The Theory of Planned Behavior questionnaire included the following constructs:

A) Patients’ Attitudes toward Eye Care: Included 9 questions; B) Patients’ Perceived Behavior Control in Eye Care: Included 5 questions; C) Patients’ Subjective Norm for Eye Care: Included 5 questions; D) Patients’ Intention for Eye Care: Included 10 questions; and E) Patients’ performance in eye care: Included 6 questions on measuring eye care behaviors.

In this study, retinopathy prevention cares included caring behaviors regarding blood sugar control, regular visits to an ophthalmologist and timely eye examinations, adherence to a medication regimen, adherence to a proper diet, and performing appropriate physical activity. The behaviors were measured by a standard questionnaire and indices including FBS and HbA1C.

HbA1C tests were conducted using a bio-system kit and chromatography method. Bio-system kits are standard kits approved by Iran’s Ministry of Health and Medical Education.

FBS is the most common test used to diagnose diabetes. The test is done in the morning, before the person has eaten. The range of normal blood glucose is between 70 to 100 mg/dl. Levels between 100 and 126 mg/dl are considered as impaired fasting glucose or pre-diabetes. Diabetes is generally diagnosed when fasting blood glucose levels are 126 mg/dl or higher [15].

In this study scoring, validity and reliability of questionnaires was done based on the similar study [14].

### Educational intervention

In this study, based on the initial need assessment (pre-test), the educational materials were prepared and educational sessions were conducted in the form of 4 sessions as follows:

The first session focused on improving patients’ awareness of diabetes, familiarity with the structure of the eye, and proper eye care.

The second session focused on improving patients’ attitudes and subjective norms, including increasing patients’ attitudes about the importance and benefits of proper eye care and the negative consequences of not caring of it.

The third session focused on perceived behavioral control, familiarizing patients with the barriers to retinopathy and improving patients’ intentions to take proper care of their eyes.

The fourth session focused on improving retinopathy preventive behaviors, including regular blood sugar measurement, adherence to a proper diet, seeing an ophthalmologist, and taking medications regularly.

Finally 3 months after the completion of the educational intervention, using the questionnaire and FBS and HbA1C, the data of both intervention and control groups were collected again and both groups were compared with each other.

### Data analysis

Data analysis was performed using SPSS version 22 and according to the normality of data distribution based on Kolmogorov-Smirnov test, the data were analyzed using Chi-Square, Pair t-test, and Independent t-test. Significance level of tests was considered less than 0.05.

### Ethical considerations

The study protocol was reviewed and approved by the ethic committee of research in Arak university of medical sciences (Approval ID: IR.ARAKMU.REC.1397.169). This trial has been registered at Iranian Registry of Clinical Trials, IRCT20180819040834N1. Written informed consent was obtained from all participants, and data are being kept confidential and anonymous.

## Results

Tables 1 and 2 presents descriptive statistics for the diabetes sample. The mean age of patients with diabetes in the intervention and control group was 57.6 ± 8 and 59.1 ± 7.1 years respectively, which did not have a significant difference based on the results of independent t-test (*p* = 0.381). Other demographic characteristics of the patients studied are reported in Tables 1 and 2. The results showed that there was no significant difference between the two groups of intervention and control in terms of TPB constructs before the intervention. After the intervention, the independent t-test showed a significant difference between the intervention and control groups in terms of TPB constructs and retinopathy preventive behaviors (Table 3). Performance of the intervention group in retinopathy-preventive behaviors increased from 2.95 ± 1.42 to 4.48 ± 1.45 after the intervention (*p* < 0.001).

**Table 1:** Comparison of the intervention and control groups, concerning the demographic variables

Group	Control		Intervention		***P***- Value
Variable	Mean	SD	Mean	SD	
Age (years)	59.1	7.1	57.6	8	**0.381**
Duration of disease (years)	9.7	4.3	6.5	4	**0.601**
Weight (Kg)	74.9	19.6	75.6	16.8	**0.662**
Height (Cm)	158.1	8.7	161.8	9.6	**0.328**
BMI (Weight/height (m^2^))	29.71	3.2	28.93	3.5	**0.098**

**Table 2:** Comparison of the intervention and control groups, concerning the demographic variables

Group	Control		Intervention		***P***- Value
Variable	Frequency (N)	Percent (%)	Frequency (N)	Percent (%)	
Sex					
Female	35	71.4	27	62.8	**0.48**
Male	14	28.6	16	37.2	
Marital status					
Married	44	91.6	35	81.4	**0.12**
Single	4	8.4	8	18.6	
Level of Educational					
Elementary	1	2	1	2.3	**0.81**
Diploma	33	67.3	31	72.1	
University	15	30.6	11	25.4	
Type of treatment					
Tablet	38	80	33	71	**0.39**
Insulin injection	7	16	9	19	
Diet	2	4	5	10

**Table 3:** Comparison of the intervention and control groups, concerning TPB before and after the intervention

Group	Control		Intervention		***P***-value^**a**^
Variable	Mean	SD	Mean	SD	
**Knowledge**					
**Before**	4.4	1.8	4.3	1.6	0.634
**After**	4.5	1.8	7.9	1.9	0.001
***P*-value**^**b**^	0.775		0.001		
**Attitude**					
**Before**	32.17	2.05	32.19	2.37	
**After**	32.64	2.42	35.56	4.30	
***P*-value**^**b**^	0.335		0.001		
**Subjective norm**					
**Before**	21.04	3.84	20.90	4.49	0.870
**After**	16.22	3.61	21.11	4.4	0.001
***P*-value**^**b**^	0.095		0.049		
**Perceived behavior control**					
**Before**	10.28	3.19	11.04	2.82	0.226
**After**	8.31	2.70	11.26	2.80	0.001
***P*-value**^**b**^	0.367		0.092		
**Behavioral intention**					
**Before**	25.78	2.76	25.5	3.8	0.80
***P*-value**^**b**^	23.53	2.8	30.2	5.6	0.001
**After**	0.137		0.001		
**Performance**					
**Before**	2.91	1.24	2.95	1.42	0.88
**After**	3.08	1.30	4.48	1.45	0.001
***P*-value**^**b**^	0.294		0.001		

^a^ Independent t test

^b^ Paired t test

As shown in Table 4, the mean FBS and HbA1C of the patients in the intervention group decreased significantly 3 months after the educational intervention (*P* < 0.05), while this decrease was not observed in the control group.

**Table 4:** Comparison of the intervention and control groups, concerning FBS and HbA1C before and after the intervention

Group	Control		Intervention		***P***-value^**a**^
Variable	Mean	SD	Mean	SD	
**FBS**					
**Before**	163.11	34.15	159.17	35.29	0.167
**After**	161.59	45.11	121.54	44.25	0.001
***P*-value**^**b**^	0.089		0.001		
**HbA1C**					
**Before**	7.36	1.43	7.41	1.44	0.872
**After**	7.23	1.32	6.54	1.29	0.005
***P*-value**^**b**^	0.099		0.043		

^a^ Independent t test

^b^ Paired t test

## Discussion

This study found that the training of patients with diabetes based on Theory of Planned Behavior, promoted preventive behaviors of ocular complications and improved control of FBS and HbA1C in the patients. In this study, the results of the pre-test showed that the patients’ information about retinopathy was very weak. While after intervention the majority of patients were aware of the positive influence of good glycemic control and of regular eye examinations by an ophthalmologist on the prevention of diabetic eye diseases.

In a study in Goa, India [5], only about one-third of patients (34%) were aware of the ocular complications of diabetes. Other similar studies conducted in Nepal [16] and Africa [17] reported that patients’ awareness was very low, and researchers said that patients need training in this area.

In a study by Bandurska et al., in Poland the level of awareness of patients with diabetic retinopathy increased from 39 to 44% due to the training given to them [18], which is consistent with the results of the present study. But in a study by Dan et al. in training with multimedia for 10 min showed a very small increase in patient awareness [19]. The reason for the failure of the above program can be one-way training and short training time, as well as the high cost of eye care.

In the present study, an increase in the attitude of patients with diabetes towards diabetes care has been reported in similar study in Iran [20] and Ontario, Canada [21]. However, there was no change in attitude in Khalaf et al. study due to the short duration of training [22] because changing patients’ attitudes, unlike their awareness, requires longer intervention. In the study of Grimshaw et al. in Ottawa Hospital Research Institute [23] and the study of Zwarenstein et al. in London [24], training by providing educational booklets to physicians to increase their attitude towards referring patients for ophthalmological examinations was not so effective. Therefore, the results of these studies are inconsistent with the present study. The method of presenting the educational booklets should not be used as the only way to teach and change the attitude just because of its low cost.

In the present study, providing the educational content in educational sessions through role-playing, expressing the role of influential people on the patient’s behaviors, providing relevant educational materials through an ophthalmologist, as well as giving educational booklets led to an increase in subjective norms. In the Woolley study on individuals with type 2 diabetes mellitus were identified through diabetes eye clinics and general practices in UK [25], physicians and nurses, in the Azami study [26], only nurses, in the Graham-Rowe study [27], health care providers and physicians were identified as sources of information and factors influencing patients’ subjective norms. This shows that training through these people can be more effective.

In the present study, presenting an educational program on factors facilitating the behaviors, providing incentives, reducing and eliminating the perceived barriers, breaking the behaviors into small steps, practical education, and using the experiences of other patients with diabetes increased the patients’ perceived behavioral control in the intervention group. Alwazae et al. reported the high cost and lack of awareness of Saudi patients as barriers to the prevention of retinopathy. In this study, the mean age of the patients was 54 and most of them (69%) were women, which is largely consistent with the demographic characteristics of the current study sample [10]. However, in the study of Hardeman et al. in UK, increased perceived behavioral control and attitudes did not lead to increased behavior in patients with diabetes. Researchers attribute this to environmental and non-behavioral factors and recommend further research in this area [28].

The results of the present study showed that the mean scores of behavioral intention in the intervention group significantly increased after the educational intervention. In Lin study, TPB-based training during the five educational sessions, along with 3 months of follow-up, had a positive effect on increasing the behavioral intention of patients regarding diabetes care behaviors [12]. Other studies have emphasized the role of behavioral intention in controlling blood sugar [11].

In the present study, the mean score of performance in the intervention group was (4.48) significantly higher than the control group after the educational intervention. Of course for the general public, some educational content can be delivered indirectly by means of educational booklets, pamphlets or via social media to reduce the number of training sessions.

Raising awareness as well as other constructs of Theory of Planned Behavior, including attitudes, behavioral intention, and perceived behavioral control, all led to increased skills and preventive behaviors by patients with diabetes. Liu and colleagues also suggest that eye care behaviors can prevent blindness by up to 90% [29]. In the Mumba study, the number of ophthalmological examinations increased from 29 to 47% by training patients at a Tanzanian referral hospital [30], but Dan et al. did not observe an increase in patients performance by providing 10-min educational multimedia [19].

In the present study, in addition to the use of educational videos, direct and face-to-face teaching methods were also used and the performance of the patients increased further and a significant change in the mean FBS and HbA1C of the patients was observed after the educational intervention. Given that HbA1c represents the mean fluctuations in blood sugar over the past 3 months, reducing it can greatly prevent the complications of diabetes, including retinopathy in patients with diabetes in Poland [18] and Ontario, Canada [21] that all of whom needed education. Face-to-face education reduced patients’ HbA1C by 48% in the Hidvégi study in Budapest [31] and by 1.5% in the Bandurska study in Poland [18].

Some limitations of this study are using a small sample and self-reported questionnaires which may be prone to recall as well as desirability bias. Moreover the attitude and performance of patients in eye care was assessed over the past 3 months, whereas longer follow-up could provide more accurate results.

Therefore, it is recommended that the educational program and the follow-up of patients be continued for a longer period of time and the outcomes be evaluated in longer periods after the intervention. Finally it is suggested that in future studies be conducted with a larger sample size.

## Conclusion

Teaching patients with diabetes based on TPB can improve the preventive behaviors of retinopathy complications and also improve the mean FBS and HbA1C in patients with diabetes. Finally, instead of using traditional methods of educating patients with diabetes, it is recommended to use educational approaches in which patients participate and are active. Using successful patients as educators, supporting patients to empower them to engage in self-care activities, and using visual media in educational programs to make the training more effective are also recommended.

## Data Availability

The datasets generated during and analyzed during the current study are available from the corresponding author.

## Abbreviations

FBS: Fasting Blood Sugar
TPB: Theory of Planned Behavior
T2DM: Type 2 Diabetes Mellitus
CVI: Content Validity Index
CVR: Content Validity Ratio
